# Safety and tolerability of nevirapine-based antiretroviral therapy in HIV-infected patients receiving fluconazole for cryptococcal prophylaxis: a retrospective cohort study

**DOI:** 10.1186/1471-2334-5-67

**Published:** 2005-08-24

**Authors:** Weerawat Manosuthi, Nopphanath Chumpathat, Achara Chaovavanich, Somnuek Sungkanuparph

**Affiliations:** 1 Bamrasnaradura Institute, Ministry of Public Health, Nonthaburi, 11000, Thailand; 2 Faculty of Medicine Ramathibodi Hospital, Mahidol University, Bangkok, 10400, Thailand

## Abstract

**Background:**

To compare the adverse events after initiation of nevirapine-based ART among HIV-infected patients who did not receive fluconazole (group A), received fluconazole 400 mg/week (group B), and received fluconazole 200 mg/day (group C).

**Methods:**

A retrospective cohort study was conducted among HIV-infected patients who began NVP-based ART between December 2003 and September 2004. Patients were followed up for 6 months. Clinical hepatitis, elevated aspartate aminotransferase (AST) and alanine aminotransferase (ALT) (> 3 times from baseline), and skin rashes were studied.

**Results:**

There were 686 patients; 225, 392, and 69 patients in group A, B, and C, respectively. Baseline characteristics including age, previous opportunistic infections, use of antituberculous drugs, and baseline aminotransferase levels among the three groups were similar. Group C had a higher proportion of men (*p *= 0.016). Baseline median (IQR) CD4 cell counts were 85 (21–159), 18 (7–48), and 16 (5–35) cell/mm^3 ^in group A, B, and C, respectively (*p *< 0.001). Of 2/225 (0.9%), 4/392 (1.0%), and 0/69 (0%) patients in group A, B, and C developed clinical hepatitis (*p *= 0.705). There were no significant difference of elevated AST or ALT among the three groups (*p *> 0.05). By logistic regression, receiving fluconazole was not predictive of clinical hepatitis, elevated aminotransferase, or skin rashes. At 6 months after initiating NVP, 174 (77.3%) patients in group A, 309 (78.8%) patients in group B, and 58 (84.1%) patients in group C remained on NVP.

**Conclusion:**

Initiation of NVP-based ART among Thais with advance HIV disease receiving fluconazole is safe and well-tolerated. nevirapine should not be contraindicated for patients receiving fluconazole for treatment or prophylaxis of cryptococcosis.

## Background

Highly active antiretroviral therapy (HAART) has been widely used for the treatment of human immunodeficiency virus (HIV) infected patients with successful immune restoration and reductions in morbidity and mortality [1,2]. However, access to antiretroviral therapy for HIV-infected patients in resource-limited countries is still a major obstacle [3,4]. HIV-infected patients in these areas often presented with advanced HIV disease. Nevirapine (NVP) is a non-nucleoside reverse transcriptase inhibitor (NNRTI) that has shown efficacy even in advanced HIV disease [5-8]. NVP-based HAART has been widely used in the resource-limited countries because of its efficacy, relative low cost, and availability. Additionally, NVP is part of two of the four World Health Organization-recommended generic combinations for the 3 × 5 program in resource-limited countries [9]. NVP-related adverse events particularly skin rashes and hepatotoxicity have been well recognized as the limitation of NVP use [10-12]. NVP-associated skin rashes usually appear after one to four weeks of treatment [10]. The risk of hepatotoxicity is greatest in the first six weeks of treatment and continues through 18 weeks of treatment [13].

Fluconazole is a commonly used for the prophylaxis and treatment of cryptococcosis in HIV-infected patients. Fluconazole 400 mg/day has been used as standard therapy for treatment of cryptocoocal meningitis after completion of two weeks of amphotericin B [12]. Fluconazole 200 mg/day is used for secondary prophylaxis until immune reconstitution occurs with HAART [14]. Fluconazole 400 mg/week is recommended for primary prophylaxis among HIV-infected patients with CD4 cell count <100 cells/mm^3 ^in Thailand and some developing countries because of a high prevalence of cryptococcosis and the evidence of a survival benefit [15]. Nonetheless, the potential drug-drug interaction between NVP and fluconazole is a major concern. Both drugs can induce cytochrome P450 isoenzymes in the liver [16-18]. Geel *et al *recently reported that fluconazole significantly raised plasma NVP levels and may cause serious hepatotoxicity [19]. So far, there have not been enough data or any recommendations to adjust NVP dosage for the concomitant use of both drugs in order to avoiding adverse events. A previous study demonstrated that genetic disposition may play a role in NVP hypersensitivity reactions [20]. There is little data of safety and tolerability for concurrent use of NVP and fluconazole in Asian populations. We therefore conducted this retrospective cohort study to compare the frequencies of hepatotoxicity and skin rashes among HIV-infected patients who received fluconazole and subsequently received NVP-based HAART regimens.

## Methods

A retrospective cohort study was conducted among antiretroviral-naïve HIV-infected patients who began a NVP-based HAART regimens between December 2003 and September 2004 at Bamrasnaradura Institute, Ministry of Public Health, Nonthaburi, Thailand. Inclusion criteria were as follows: (1) HIV-infected patients > 15 years of age, (2) naïve to antiretroviral therapy, (3) began antiretroviral therapy with a NVP-based HAART regimen, (4) used NVP 200-mg once-daily lead-in dose, prior to escalation to 200 mg twice daily. Each eligible patient was classified into one of three groups according to whether he or she received fluconazole or not and the different dosages of fluconazole as follows: not received fluconazole (group A), received fluconazole 400 mg/week (group B), and received fluconazole 200 mg/day (group C). All patients were followed for 6 months after initiating NVP-based HAART. The primary objective was to compare the frequency of clinical hepatitis among the three groups. The secondary objectives of interest were to compare the frequencies of elevated aspartate aminotransferase (AST) and alanine aminotransferase (ALT) > three times from baseline at six months after initiation of NVP and to compare the frequencies skin rashes among the three groups.

The patients' clinical statuses including clinical hepatitis and skin rashes were assessed monthly by the attending physician. Liver functions test was performed at 6 months after initiating NVP in case of no evidence of hepatitis. Clinical hepatitis was defined as symptomatic hepatitis and either AST or ALT elevated > five times the upper limit of normal. Signs and symptoms of hepatitis may include anorexia, malaise, jaundice, nausea/vomiting, hepatomegaly, and hepatic tenderness. Skin rash was determined by medical record of skin reactions including diffuse erythematous or maculopapular rash, urticarial rash, serum sickness-like reaction, Stevens-Johnson syndrome, and toxic epidermal necrolysis.

Mean (± standard deviation, SD), median (interquartile range, IQR) and frequencies (%) were used to describe patients' characteristics in each treatment groups. Chi-square test and Kruskal-Walis test were used to compare categorical and continuous variables among the treatment groups, respectively. Logistic regression was used to determine predicting factors for each adverse event of interest. The possible risk factors were entered into the model of binary logistic regression. Spearman correlation was use to study the relationship between skin rashes and clinical hepatitis, skin rashes and aminotransferase levels. All analyses were performed using SPSS program version 11.5. A *P *value less than 0.05 was considered statistically significant. The study was approved from the institute review board.

## Results

A total of 686 patients were eligible and included in the study. Of 686 patients; 225, 392, and 69 patients were classified into group A, B, and C, respectively. The baseline characteristics of patients prior to initiation of HAART are described and compared among the three groups as shown in Table 1. Baseline characteristics including age, previous major opportunistic infections (OIs), use for antituberculous drugs therapy, baseline AST and ALT, and baseline HIV RNA prior to HAART were not different among the three groups. Group C patients had a greater proportion of men than the other groups (*p *= 0.016). Group B and group C patients had more advanced HIV disease [i.e., lower body weight (*p *= 0.008), lower baseline CD4 cell counts prior to initiation of HAART (*p *< 0.001), and higher proportion receiving co-trimoxazole(*p *< 0.001)] than patients in group A.

**Table 1:** Baseline characteristic of 686 patients receiving NVP-based HAART.

**Baseline characteristics**	**Not receiving fluconazole**	**Receiving fluconazole**		***P *value**
			
	*(n = 225)*	**400 mg/week ***(n = 392)*	**200 mg/day ***(n = 69)*	
Gender: Male	116 (51.6%)	213 (54.3%)	49 (71.0%)	0.016
Female	109 (48.4%)	179 (45.7%)	20 (29.0%)	
Age, years, mean ± SD	36.0 ± 7.5	35.0 ± 7.4	34.7 ± 8.03	0.200
Weight, kg., mean ± SD	54.9 ± 10.6	52.3 ± 10.0	51.4 ± 8.7	0.008

*Previous opportunistic infections*, patients (%)				
Any major opportunistic infections	53 (23.6%)	120 (30.6%)	24 (34.8%)	0.368
Tuberculosis	32 (14.2%)	64 (16.3%)	17 (24.6%)	0.124
PCP	31 (13.8%)	47 (12.0%)	8 (11.6%)	0.787
Histoplasmosis	3 (1.3%)	1 (0.3%)	-	0.190
Penicilliosis	3 (1.3%)	0 (0.0%)	-	0.046
Toxoplasmosis	-	5 (1.3%)	-	0.151
MAC	2 (0.9%)	2 (0.5%)	-	0.669

*Concurrent medication*				
Cotrimoxazole	176 (78.2%)	363 (93.3%)	57 (82.6%)	< 0.001
Antituberculous drugs	47 (20.9%)	82 (20.9%)	22 (31.9%)	0.095

Baseline CD_4 _cell count, cell/mm^3^, median (IQR)	85 (21–159)	18 (7–48)	16 (5–35)	< 0.001
Baseline % CD4, median (IQR)	7 (2–11)	2 (1–5)	2 (1–5)	< 0.001
Baseline plasma HIV RNA,	207,500	270,000	169,000	0.095
copies/ml, median (IQR)	(68,375–549,500)	(118,000–545,000)	(76,750–415,000)	
Baseline AST, U/L, median (IQR)	31 (23–46.5)	32 (25–53.2)	34 (24–53)	0.358
Baseline ALT, U/L, median (IQR)	23 (17–39)	28 (17–45)	32 (17–49.5)	0.177
Baseline TB, mg/dl, median (IQR)	0.5 (0.4–0.65)	0.5 (0.4–0.7)	0.5 (0.4–0.8)	0.843

One hundred and four of 255 (40.8%) in group A, 188 of 392 (48.0%) in group B, and 36 of 69 (52.2%) in group C had AST testing at 6 months after initiating NVP. Seventy one of 255 (29.4%), 95 of 392 (24.2%), and 22 of 69 (31.9%) in corresponding groups had ALT testing at 6 months after initiating NVP. The prevalence of patients who developed adverse events including clinical hepatitis, AST and ALT increase > 3 times from baseline, and skin rashes are shown in Table 2. The prevalence of clinical hepatitis, elevated AST, and elevated ALT were not different among the three groups (*p *> 0.05). There was a significant difference among the groups in the prevalence of skin rashes (*p *= 0.016).

**Table 2:** Frequency of adverse events among the three groups.

**Adverse events**	**Not receiving fluconazole**	**Receiving fluconazole**		***P *value**
			
	*(n = 225)*	**400 mg/week ***(n = 392)*	**200 mg/day ***(n = 69)*	
Clinical hepatitis	2/225 (0.9%)	4/392 (1.0%)	0/69 (0%)	0.705
Elevated AST > 3 times from baseline	3/104 (2.9%)	5/188 (2.7%)	3/36 (8.3%)	0.192
Elevated ALT > 3 times from baseline	6/71 (8.5%)	7/95 (7.4%)	2/22 (9.1%)	0.938
Skin Rash	23/225 (10.2%)	27/392 (6.9%)	0/69 (0%)	0.016

Results of logistic regression analysis indicated that receiving anti-tuberculous drugs was a significant predictor of clinical hepatitis (*p *= 0.025) (Table 3). Gender, body weight, baseline CD4 cell counts, or receiving fluconazole were not predictive of clinical hepatitis. Logistic regression analyses of models including gender, body weight, baseline CD4 cell count, receiving anti-tuberculous drugs, and receiving fluconazole, were not significant predictors of elevated AST > 3 times from baseline (χ^2 ^(5, N = 328) = 3.72, *p *= 0.592) and elevated ALT > 3 times from baseline (χ^2 ^(5, N = 188) = 7.26, *p *= 0.298). Logistic regression analysis of skin rashes indicated that receiving anti-tuberculous drugs was a significant predictor of skin rashes (Table 4). Gender, body weight, baseline CD4 cell counts, or receiving fluconazole were not predictive of skin rashes.

**Table 3:** Logistic regression of possible risk factors and clinical hepatitis

**Risk factors**	**Odd Ratios**	**95% confidence interval**	***P *value**
Female gender	1.25	0.21–7.49	0.809
Body weight	1.08	0.98–1.17	0.076
Baseline CD4 cell count	0.99	0.97–1.01	0.352
Receiving anti-tuberculous drugs	7.42	1.27–43.31	0.026
Receiving fluconazole	1.12	0.18–6.90	0.901
Receiving Cotrimoxazole	0.84	0.21–5.44	0.675

**Table 4:** Logistic regression of possible risk factors and skin rashes.

**Factors**	**Odd Ratios**	**95% confidence interval**	***P *value**
Female gender	1.26	0.67–2.39	0.470
Body weight	1.02	0.98–1.05	0.347
Baseline CD4 cell count	1.00	0.99–1.01	0.873
Receiving anti-tuberculous drugs	2.96	1.04–8.47	0.043
Receiving fluconazole	1.56	0.79–3.08	0.201
Receiving Cotrimoxazole	1.21	0.68–2.91	0.594

The results of correlation analyses show that there was no correlation between skin rashes and clinical hepatitis (r_s _= 0.026, *p *= 0.491), skin rashes and AST (r_s _= 0.003, *p *= 0.948), or skin rashes and ALT (r_s _= 0.034, *p *= 0.637). The tolerability of NVP-based HAART is shown in Table 5. Regarding NVP-associated skin rashes, the majority (> 90%) of patients developed diffuse maculopapular rashes. Only 1 of 25 patient in group A and 2 of 26 patients in groups B developed Steven-Johnson syndromes. Nine patients died, 1 in group A and 8 in group B. The majority of patients died from opportunistic infections (i.e., 2 cryptococcosis, 2 bacterial pneumonitis, 1 *Mycobacterium avium complex *infection, and 3 wasting syndromes). No patient died from hepatitis.

**Table 5:** Tolerability of NVP-based HAART among the three groups.

**Tolerability outcomes**	**Not receiving fluconazole**	**Receiving fluconazole**		***P *value**
			
	*(n = 225)*	**400 mg/week ***(n = 392)*	**200 mg/day ***(n = 69)*	
On NVP-based HAART at six months	174 (77.3%)	309 (78.8%)	58 (84.1%)	0.532
Reasons of discontinuation				0.448
Adverse events	26 (11.5%)	29 (7.4%)	3 (4.3%)	
Skin rashes	25/26 (96.2%)	26/29 (89.7%)	3 of 3 (100.0%)	
Hepatotoxicity	1/26 (2.8%)	3/29 (10.3%)	-	
Death	1 (0.4%)	8 (2.0%)	-	
Loss to follow-up	20 (8.9%)	39 (9.9%)	7 (10.1%)	
Refer	4 (1.8%)	7 (1.8%)	1 (1.4%)	

## Discussion

In the present study, we have shown that the prevalence of clinical hepatitis and AST and ALT elevation 6 months after initiating HAART among HIV-infected patients who concurrently received NVP and fluconazole were not different from those who received NVP- based HAART without fluconazole. These findings suggest that administration of NVP-based HAART regimens in HIV-infected patients who receive fluconazole dose not increase the risk of hepatotoxicity. The prevalence of clinical hepatitis and aminotransferase elevations in our study was lower than that reported in studies conducted in African HIV-infected patients [19,20]. There may be several explanations for this discrepancy. First, the patients in this study had very low CD4 cell counts (i.e., 97% had CD4 cell counts < 200 cells/mm^3 ^and 80% had CD4 cell counts < 100 cells/mm^3^). High CD4 cell count is a predictor of hepatotoxicity [11,19]. Second, there was a higher proportion of men in our study than the previous studies [17,18]. CD4 cell counts are more predictive of hepatotoxicity among men than women (i.e., men who have a CD4 cell counts > 400 cells/mm^3 ^are at a greater risk of hepatitis than women with CD4 cell counts > 250 cells/mm^3^) [13,21]. Third, our study included Asians, which was different from the previous study [11,17,18]. Sanne *et al *reported that African women may have an increased likelihood of hepatotoxicity [20]. The previous studies demonstrate that there are differences in drug and alcohol metabolism that may be related to genetic or environmental factors (e.g., differences in diet and protein intake) [22-24]. There is little data on differences in NVP metabolism based on genetic disposition.

While logistic regression analyses in the present study show that receiving fluconazole was not predictive for any adverse events, receiving anti-tuberculous drugs was significantly predictive for both clinical hepatitis and skin rashes. The patients in our large cohort had overall 16% of concurrent tuberculosis and the results from the regression analyses can demonstrate this important issue. These findings point out that concurrent concomitant use of NVP and anti-tuberculous drugs may a cause of more concern. We suggest to closely monitoring liver function test in patients with concomitant use of NVP and anti-tuberculous drugs. Further well-designed prospective study is needed.

Regarding NVP-associated skin rashes, the overall prevalence in the present study was similar to previous study in Thai patients with advanced HIV disease [25]. The incidence of NVP-associated skin rashes in the patients who received Fluconazole was lower than the other groups. This may be explained by the lower CD4 cells count in this group. As the previous studies have demonstrated that persons with a higher baseline CD4 cell counts are likely to develop rash from NVP [26]. Given there was no correlation between skin rashes and clinical hepatitis or elevated aminotransferase, these two entities of adverse events may need different approaches for monitoring and management.

Overall about 80% of the patients in our cohort continued on NVP-based HAART at six months. Although all patients were initiated with NVP 200-mg once-daily lead-in dose prior to escalation to 200 mg twice daily, skin rash was still the major adverse event led to discontinuation of NVP-based HAART. The previous studies using short-course prednisolone were failed to prevent NVP-associated skin rashes [26,27]. The further study to minimize this major limitation is needed.

NVP-based HAART is a common regimen which widely used for treatment of HIV-infected patients in resource-limited countries due to its affordability. Until the other options are more accessible, NVP-based HAART is still a key regimen to scale up treatment of HIV in resource-limited countries. Our study may provide the safety data of concomitant use of NVP and fluconazole and support the use of NVP in patients receiving fluconazole for treatment or prophylaxis of cryptococcal diseases.

The limitation of the present study is the nature of retrospective study that patients' clinical status may be underreported. First, not all patients had been checked for aminotransferase level during the first 6 months. Only 328 and 188 patients had been checked for AST and ALT levels, respectively. However, we had analyzed the demographics and adverse events between patients who had and did not have AST or ALT levels and found that there were no statistically significant differences. We did not have data of plasma NVP level in this cohort due to retrospective nature. However, Dailly *et al *reported that there was no association between plasma NVP level and hepatotoxicity [28]. Ultimately, the present study did not assess patients who initiated NVP while received fluconazole 400 mg/day.

## Conclusion

In summary, receiving of fluconazole for treatment or prophylaxis of cryptococcosis does not increase the risk of hepatotoxicity, elevated aminotransferase, or skin rashes from NVP. Initiation of NVP-based HAART among HIV-infected patients receiving fluconazole is safe and well-tolerated. Use of anti-tuberculous drugs is predictive of clinical hepatitis and skin rashes. Initiation of NVP-based HAART in HIV-infected patients receiving anti-tuberculous needs closely monitoring and prompt management.

## List of abbreviations

HAART: Highly active anti-retroviral therapy, HIV: Human immunodeficiency virus, NVP: Nevirapine, NNRTI: Non-nucleoside reverse transcriptase inhibitor, AST: Aspartate aminotransferase, ALT: Alanine aminotransferase

## Competing interests

The author(s) declare that they have no competing interests.

## Authors' contributions

WM participated in the design of the study. NC performed statistical analysis. AC participated in the design of the study. SS participated in the design of the study and statistical analysis.

## Pre-publication history

The pre-publication history for this paper can be accessed here:


